# Explainable artificial intelligence in breast cancer detection and risk prediction: A systematic scoping review

**DOI:** 10.1002/cai2.136

**Published:** 2024-07-03

**Authors:** Amirehsan Ghasemi, Soheil Hashtarkhani, David L. Schwartz, Arash Shaban‐Nejad

**Affiliations:** ^1^ Department of Pediatrics, Center for Biomedical Informatics, College of Medicine University of Tennessee Health Science Center Memphis Tennessee USA; ^2^ The Bredesen Center for Interdisciplinary Research and Graduate Education University of Tennessee Knoxville Tennessee USA; ^3^ Department of Radiation Oncology, College of Medicine University of Tennessee Health Science Center Memphis Tennessee USA

**Keywords:** breast cancer, deep learning, explainable artificial intelligence, interpretable AI, machine learning, XAI

## Abstract

With the advances in artificial intelligence (AI), data‐driven algorithms are becoming increasingly popular in the medical domain. However, due to the nonlinear and complex behavior of many of these algorithms, decision‐making by such algorithms is not trustworthy for clinicians and is considered a black‐box process. Hence, the scientific community has introduced explainable artificial intelligence (XAI) to remedy the problem. This systematic scoping review investigates the application of XAI in breast cancer detection and risk prediction. We conducted a comprehensive search on Scopus, IEEE Explore, PubMed, and Google Scholar (first 50 citations) using a systematic search strategy. The search spanned from January 2017 to July 2023, focusing on peer‐reviewed studies implementing XAI methods in breast cancer datasets. Thirty studies met our inclusion criteria and were included in the analysis. The results revealed that SHapley Additive exPlanations (SHAP) is the top model‐agnostic XAI technique in breast cancer research in terms of usage, explaining the model prediction results, diagnosis and classification of biomarkers, and prognosis and survival analysis. Additionally, the SHAP model primarily explained tree‐based ensemble machine learning models. The most common reason is that SHAP is model agnostic, which makes it both popular and useful for explaining any model prediction. Additionally, it is relatively easy to implement effectively and completely suits performant models, such as tree‐based models. Explainable AI improves the transparency, interpretability, fairness, and trustworthiness of AI‐enabled health systems and medical devices and, ultimately, the quality of care and outcomes.

## Graphical abstract

This systematic review is carried out using the preferred reporting items on systematic reviews and meta‐analysis (PRISMA) guideline in three steps as follows:

Step 1:
Identifying studies (total of 193 studies were initially included).
Step 2:
Selecting the studies (total of 30 articles met the inclusion criteria for our comprehensive review).
Step 3:
Data extraction and summarization (A data extraction form was developed in Google Sheets, consisting of eight variables, including Authors, Year, the aim of the study (Objective), Data set(s), Data type, Important features, type of artificial intelligence (machine learning or deep learning), and the Explained model. Two reviewers (Amirehsan Ghasemi and Soheil Hashtarkhani) extracted data from all included articles, and any disagreement was resolved by consensus.

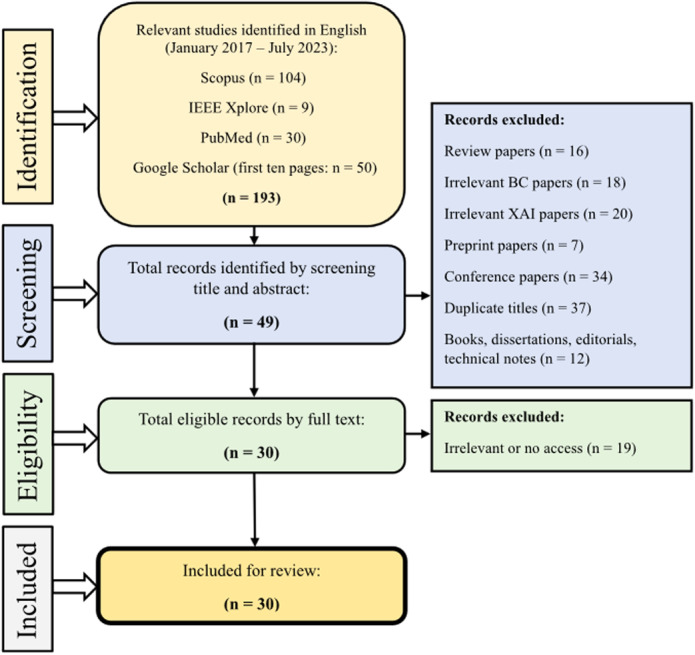

**Abbreviations**
AI: artificial intelligence
BC: breast cancer
CAD: computer‐aided diagnosis
CAM: class activation map
CNN: convolutional neural network
CPH: cox proportional hazards
CuMiDa: curated microarray database
DBT: digital breast tomosynthesis
DDSM: digital database for screening mammography
DL: deep learning
ET: extra‐trees
FL: federated learning
GAP: global average pooling
GCN: graph‐CNN
GEO: gene expression omnibus
GLCM: gray‐level co‐occurrence matrix
GLRP: graph layerwise relevance propagation
HITL: human‐in‐the‐loop
IDEs: invasive disease events
IHC: immunohistochemical
LIME: local interpretable model agnostic explanations
LRP: layerwise relevance propagation
MBC: male breast cancer
MIAS: Mammographic Image Analysis Society
MICW: Medical College of Wisconsin
ML: machine learning
NAC: neoadjuvant chemotherapy
NST: neoadjuvant systemic therapy
PBMC: peripheral blood mononuclear cells
PRISMA: preferred reporting items on systematic reviews and meta‐analysis
SHAP: SHapley Additive exPlanations
TCGA: The Cancer Genome Atlas
TME: tumor microenvironment
WSI: whole‐slide images
XAI: explainable artificial intelligence

## 1 INTRODUCTION

Breast cancer (BC) is one of the most common cancers with high morbidity and mortality globally. Early detection and treatment significantly increase the chances of survival [1]. With the growing interest in artificial intelligence (AI), computer‐aided diagnosis (CAD) based on AI has become a valuable tool for the detection, classification, and diagnosis of cancer biomarkers and morphological features.

Compared to rule‐based systems [2] that require human intervention in decision‐making, AI models can learn from medical data and generate new patterns by themselves. However, AI systems are susceptible to several biases [3], mostly stemming from low‐quality datasets, faulty algorithms, and human cognitive biases that may lead to inaccurate decisions, predictions, or inferences [4]. Additionally, many AI systems have raised concerns among clinicians about accountability, fairness of AI algorithms, and lack of transparency [5], a critical factor for high‐stakes domains such as healthcare, where a minor error in decision‐making can lead to irreparable consequences [6].

Despite significant progress over the last years in terms of fine‐tuning [7] and optimizing [8, 9, 10, 11] AI algorithms to tackle supervised and unsupervised tasks, a considerable number of these algorithms remain enigmatic, classified as Black‐box and are yet to be demystified. To this end, the scientific community has started investigating techniques and methods to make AI algorithms more understandable, explainable, and interpretable. In recent years, explainable artificial intelligence (XAI), coined by DARPA [12], has emerged as a notable and noteworthy topic of discussion in the AI community. The rationale behind XAI lies in the assumption that such techniques establish rules for more trustworthy AI systems by making them more transparent, understandable, interpretable, safe, and reliable while making a decision or recommending an action [13, 14].

**Figure 1:**
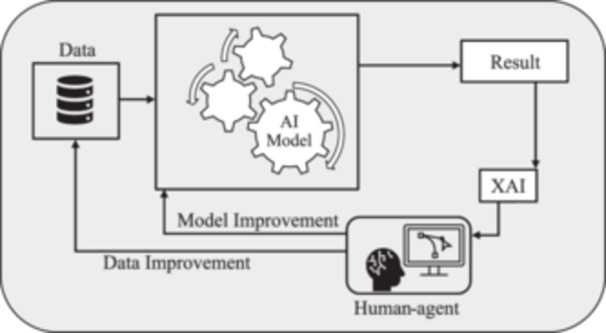
Explainable artificial intelligence (XAI) as a human‐agent problem‐solving method.

Typically, AI models are evaluated based on their prediction errors [15] without providing enough transparency to the end users throughout this process. As Figure 1 shows, XAI methods are formulated to be applied to the result and provide transparency; however, the human‐in‐the‐loop (HITL) concept must also be applied to achieve trustworthiness. XAI is basically employing human–agent interaction methods to utilize human knowledge and intuition to comprehend the rationale behind the results it gains [16]. As Figure 1 illustrates, a result generated by the AI model passes through a suitable XAI method. Then, the human agent benefits from the transparency created by the XAI to validate, confirm, or enhance the predictions [17]. It should be noted that the human agent's decision is based on the collaboration between clinicians and XAI experts. If the results are not correct or satisfying, the XAI module investigates the model, data, or both and reruns the outcomes to the AI system throughout an iterative process until a consensus over the results is reached.

Most of the review articles on XAI models investigate the application of XAI in general healthcare [18, 19, 20]. Although there have been reviews of the subject on other types of cancer [21], to the best of our knowledge, our contribution to reviewing existing XAI technologies in breast cancer screening, risk detection, and prediction is distinctive both in terms of scope and breadth. This paper provides a comprehensive summary of published studies and then elaborates on the background and concepts associated with the XAI methods used in these studies. Finally, we highlight the most popular XAI methods and explain the rationale behind their popularity.

The remainder of this paper is organized as follows. Section 2 covers the background, including the introduction to AI models, the concept of accuracy‐explainability trade‐off, and the classification of XAI methods. Section 3 describes the research method. Section 4 provides tabulated results of implemented XAI methods in breast cancer research. Section 5 discusses the details and elaborates on the utilized methods. Finally, Section 6 concludes our survey by highlighting XAI's achievements, strengths, and limitations and discussing future research opportunities.

## 2 BACKGROUND

### 2.1 Overview of AI models

AI models employ data‐driven algorithms to reach decisions or identify explanatory patterns. Machine learning (ML) algorithms fall into three types: regression and classification, which are supervised, and clustering, which is unsupervised. If the output is a continuous variable, we deal with regression, but when it is discrete labels or categories, then we use classification [22]. Clustering algorithms identify and group similar data points based on their characteristics. The most popular ML models used in breast cancer studies are listed in Table 1.

**Table 1:** List of popular machine learning (ML) models.

ML model	Acronym	Type of learning	Type of problem
Linear regression	N/A	Supervised	Regression
Logistic regression	LR	Supervised	Classification
Decision trees	DT	Supervised	Regression, classification
K‐means	N/A	Unsupervised	Clustering
Naive Bayes	NB	Supervised	Classification
Support vector machines	SVM	Supervised	Regression, classification
K‐nearest neighbors	KNN	Supervised	Regression, classification
Ensemble learning models^a^			
Extremely randomized trees [23]	Extra‐trees (ET)	Supervised	Regression, classification
Random forests [24]	RF		
Gradient boosting machines	GBM		
eXtreme gradient boosting [25]	XGBoost		
Light gradient boosting machine [26]	LightGBM		
Gradient boosted decision trees	GBDT		
Adaptive boosting [27]	AdaBoost		
Category boosting [28]	CatBoost		

a Ensemble learning is a meta‐learning approach that combines multiple models to make a decision, typically in supervised ML tasks [29].

Deep learning (DL) is a subcategory of ML that may be supervised or unsupervised. Unlike traditional machine learning, DL models require much less manual human intervention since they automate the feature extraction, saving time and resources. DL models are capable of addressing model accuracy and performance for unstructured data such as speech, images, videos, or texts, whereas classical ML models do not function effectively.

In BC datasets, we usually deal with high‐dimensional, multimodal [30] structured and unstructured data, which are often big, noisy, and sparse, making them challenging to analyze. Thanks to neural networks' universal approximation [31, 32] and the advantage of auto‐differentiation [33], deep learning models can be applied to many of these problems. DL models can learn the complex nonlinear relationships between the features and target variables, making them viable data‐driven models that enable new discoveries in breast cancer classification and detection. Frequently used DL models and their variants in BC studies are listed in Table 2.

**Table 2:** List of popular deep learning (DL) models.

DL model	Acronym	Variants	Acronym
Convolutional neural network [34]	CNN	Visual geometry group [35]	VGG (VGG‐16, VGG‐19)
		AlexNet [36]	N/A
		Xception [37]	N/A
		GoogLeNet [38]	N/A
		GoogLeNet inception V3 [39]	Inception V3
		GoogLeNet inception V4 [40]	Inception V4
		Residual networks [41]	ResNet
		ResNeXt [42]	N/A
		ResNet (Split attention networks) [43]	ResNeSt
		U‐Net [44]	N/A
		Graph convolutional network [45]	GCN
		Dense convolutional network [46]	DenseNet
		EfficientNet [47]	N/A
		MobileNet [48, 49, 50]	N/A
		ShufflieNet [51]	N/A
		SqueezeNet [52]	N/A
Recurrent neural network	RNN	Long short‐term memory [53]	LSTM
		Bidirectional LSTM [54]	BiLSTM
		Gated recurrent unit [55]	GRU

### 2.2 Accuracy‐explainability trade‐off

**Figure 2:**
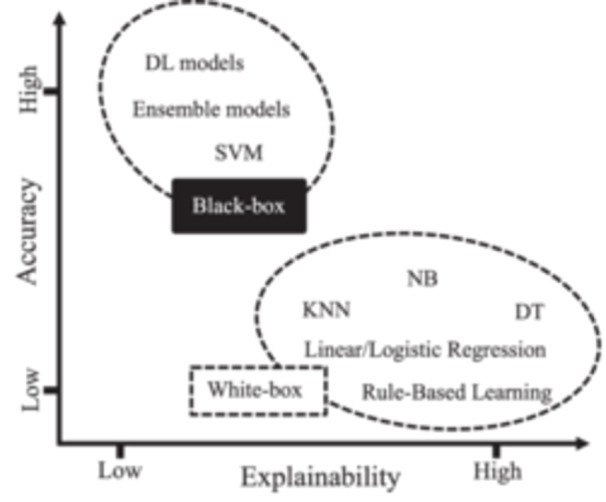
Trade‐off between model accuracy and explainability.

As Figure 2 illustrates, the accuracy‐explainability trade‐off refers to the balance between the accuracy of an AI model and its explainability [56]. The goal of any AI model is to generate highly accurate results. From the XAI perspective, the models must be explainable. However, achieving both accuracy and explainability is far from trivial. Regarding explainability, AI models can be black‐box, white‐box, or gray‐box [12, 57], as depicted in Figure 2.

White‐box models are intrinsically transparent and explainable [58]. However, they are limited to learning only linear associations between input features and the target variable. Although white‐box models may not achieve high accuracy levels, they offer human‐understandable explanations. In contrast, black‐box models are nontransparent by nature [59]. While these models may have outstanding performance, they suffer from a lack of explainability. Gray‐box models strike a balance between accuracy and explainability. Generally, any data‐driven learning algorithm, including black‐ and‐white‐box models, is considered a gray box [57, 60]. For a gray‐box model, connections from input data to model output can be explained despite not being fully transparent [60].

### 2.3 Classification of XAI methods

**Figure 3:**
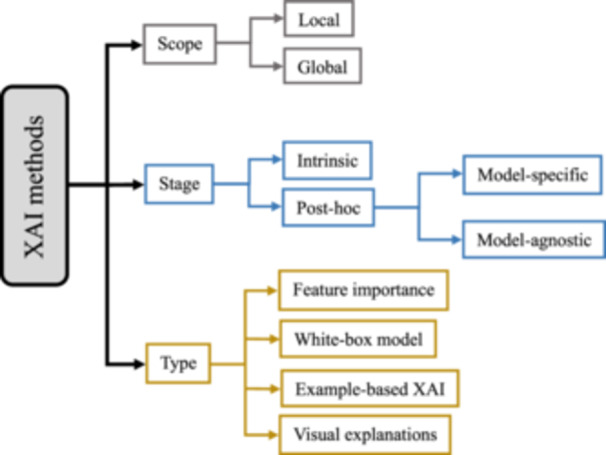
Classification of explainable artificial intelligence (XAI) methods.

The transparency of AI systems can be addressed from different perspectives. The results of XAI methods can be presented in various ways, including numerical, rules, textual, visual, or a combination of these [61]. As Figure 3 depicts, three critical factors for categorizing XAI methods of explanation exist: scope, stage, and type.

The **scope** of explainability is either local or global. The local method aims to shed light on an AI model for a specific input [62, 63] and explains why a particular decision was made by highlighting the input feature influencing the model's output. However, this approach cannot find a general relationship between input features and outputs [64]. The global method provides a broader understanding by analyzing the model's overall structure and general patterns across the entire data set or a larger subset, helping users understand its biases, limitations, and general decision‐making patterns [62, 63].

Intrinsic and post hoc [65] refer to the **stage** of explanation. The intrinsic approach refers to using white‐box models which are interpretable by nature. The post hoc approach relates to explainable methods that “explain the model predictions after the training and inference processes” [62, 64]. Generally, the post hoc approach, compared to intrinsic models (white‐box models) [65], is more accurate since post hoc explainable methods must be applied to black‐box models' prediction, and black‐box models tend to perform better in results. Although some of these methods, such as rule extraction [66] and tree extraction [67], can turn black‐box models into white‐box, there is a complexity–accuracy trade‐off [57, 64]. Additionally, post hoc methods are either model‐specific or model‐agnostic. Model‐specific methods are designed to explain specific black‐box models by investigating their internal factors and interactions [64]. For example, many techniques are developed to analyze DL models, which attempt to find the contribution of artificial neurons on their final decisions through backpropagation (backprop) error [68, 69, 70, 71]. Model‐agnostic methods provide explanations independent of a specific AI model. Some common post hoc XAI methods are tabulated in Appendix A.

XAI methods can be classified based on the **type** of explanations they offer [64]. As Figure 3 demonstrates, there are four types of explanations: feature importance, white‐box model, example‐based XAI, and visual explanations [64]. For the first type, XAI methods create numbers/values for the input features to express the feature's importance. For the second type, XAI methods “create a white‐box model that mimics the original black‐box model and is inherently explainable” [64]. The example‐based type, also known as data point [65], uses samples from the training datasets to explain the model's action. For the last type, XAI methods offer a type of explainability based on purely visual explanations [64].

## 3 RESEARCH METHOD

**Figure 4:**
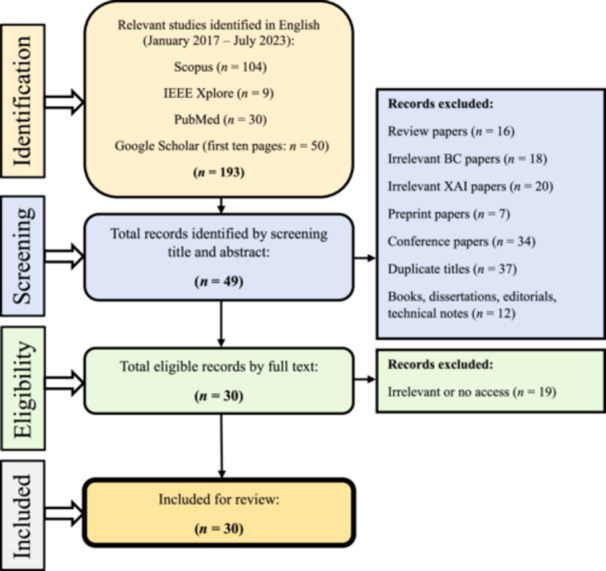
Preferred reporting items on systematic reviews and meta‐analysis guideline for article selection.

This systematic review is carried out using the preferred reporting items on systematic reviews and meta‐analysis (PRISMA) [72] guideline in three steps as follows:

**Step 1: Identifying studies**—As mentioned in the introduction, our focus for this paper was on studies that examined existing XAI methods in breast cancer research. We conducted a comprehensive search utilizing some of the most popular and trusted citation platforms [73], including Scopus, IEEE Xplore, PubMed, and Google Scholar (first 50 citations) from January 2017 to July 2023 using the combination of keywords and MeSH terms described in Table 3. A total of 193 studies were included in this step.
**Step 2: Selecting the studies**—In Step 2, we selected articles for inclusion based on specific criteria: they had to be original studies published in peer‐reviewed English‐language journals, utilizing at least one XAI methodology within the context of breast cancer. Two reviewers (Amirehsan Ghasemi and Soheil Hashtarkhani), screened citations by title and abstract, excluding various types of irrelevant papers, such as different review papers (*n* = 16), those discussing XAI but unrelated to breast cancer (*n* = 18), those discussing breast cancer but unrelated to XAI (*n* = 20), preprints awaiting peer review (*n* = 7), conference papers (*n* = 34), duplicate titles (*n* = 37), and nonresearch materials like books, dissertations, editorials, and technical notes (*n* = 12), resulting in the exclusion of 144 studies. Subsequently, 49 articles underwent full‐text scrutiny, with inaccessible or irrelevant articles being excluded. This left us with 30 articles that met the inclusion criteria for our comprehensive review. Figure 4 illustrates a summary of our search strategy and steps.
**Step 3: Data extraction and summarization**—A data extraction form was developed in Google Sheets, consisting of eight variables, including authors, year, the aim of the study (objective), data set(s), data type, important features, type of AI (ML or DL), and the explained model. Two reviewers (Amirehsan Ghasemi and Soheil Hashtarkhani) extracted data from all included articles, and any disagreement was resolved by consensus.

**Table 3:** Explored databases and the results.

	Boolean search strings	Number of search results
Database		
Scopus	TITLE‐ABS‐KEY (“Explainable Artificial Intelligence” OR “Explainable AI” OR “XAI” OR “Explainable Machine Learning” OR “Interpretable Machine Learning” OR “Interpretable AI”) AND TITLE‐ABS‐KEY (“Breast Cancer”) AND PUBYEAR > 2016	*n* = 104
IEEE Xplore	(“Abstract”:“Explainable Artificial Intelligence” OR “Abstract”:“Explainable AI” OR “Abstract”:“XAI” OR “Abstract”:“Explainable Machine Learning” OR “Abstract”:“Interpretable Machine Learning” OR “Abstract”:“Interpretable AI”) AND (“Abstract”:“Breast Cancer”)	*n* = 9
PubMed	(“Breast Neoplasms”[Mesh] OR “breast cancer”) AND (“XAI” OR “Interpretable Machine Learning” OR “Explainable Artificial Intelligence” OR “Explainable AI”) string from 2017	*n* = 30
Web search engine		
Google Scholar	(“Explainable Artificial Intelligence” OR “XAI” OR “Explainable AI”) AND “Breast Cancer”	*n* = 50

## 4 RESULTS

**Figure 5:**
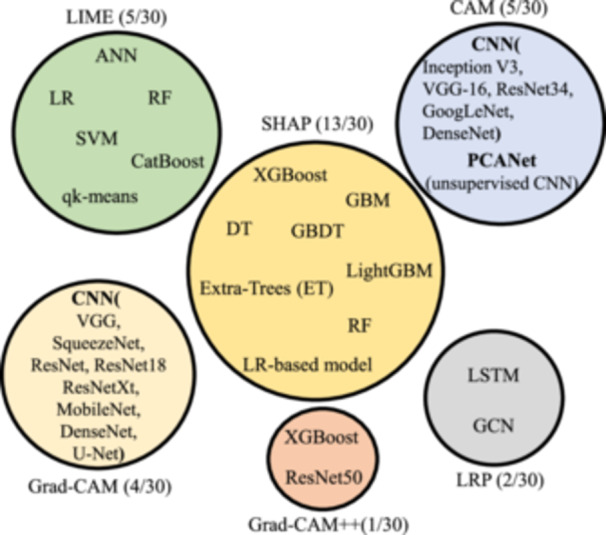
Explainable artificial intelligence (XAI) methods and explained models used in the literature.

Almost 30 studies have been identified in the literature utilizing XAI methods in breast cancer settings. XAI methods used in these studies include SHAP, LIME, CAM, Grad‐CAM, Grad‐CAM++, and LRP. The number of studies and explained AI models for each method are shown in Figure 5. Tables 4, 5, 6, 7, 8, 9 provide tabular representations of the included studies. A detailed description of the results is provided in the discussion section (Section 5).

**Table 4:** List of studies that used SHapley Additive exPlanations.

Authors	Year	Objective	Data set(s)	Data type	Important features	ML/DL	Explained model
Chakraborty et al. [74]	2021	Investigate the relationship between immune cell composition in the tumor microenvironment (TME) and the ≥5‐year survival rates of breast cancer patients	Patient clinical information for TCGA breast invasive carcinoma data from two projects on the cbioPortal	Clinical data	B cells, CD$8^{+}$ T cells, NK T cells, M0 macrophages	ML	XGBoost
Moncada‐Torres et al. [75]	2021	The Cox Proportional Hazards (CPH) (Identifying the prognostic factors that have an impact on patients' recurrence or survival)	Netherlands Cancer Registry (NCR) 36,658 non‐metastatic breast cancer patients	Text	Age, pts, ptmm	ML	XGBoost
Rezazadeh et al. [76]	2022	Breast cancer diagnosis based on the gray‐level co‐occurrence matrix (GLCM)	Data set of breast ultrasound images [77]	Ultrasound	GLCM texture features	ML	Decision tree Ensemble model (DT, GBDT, LightGBM)
Nahid et al. [78]	2022	Classify BC patients and non‐BC patients through regular examination of a few health‐related issues such as the level of Glucose, Insulin, HOMA, Leptin, etc	Data set by the University Hospital Centre of Coimbra. 116 participants (64 BC, 52 non‐BC)	Text (blood test data)	Glucose	ML	GBM
Yu et al. [79]	2022	Clarify the radiation dose‐volume effect of radiation therapy to avoid radiation‐induced lymphopenia	589 patients with breast cancer who underwent radiation therapy at the University of Hong Kong‐Shenzhen Hospital	Clinical data	Baseline lymphocyte counts protect against while the baseline hemoglobin level impacts the event of radiation‐induced lymphopenia	ML	XGBoost
Meshoul et al. [80]	2022	Improve the multiclassification performance of ML models for BC cancer subtyping for high dimensional datasets with a minimal number of instances	The Cancer Genome Atlas (TCGA)	Omics data	DNA, RNA, CNV	ML	Extra‐Trees (ET)
Kumar et al. [81]	2023	Identify potential diagnostic biomarkers for BC	NCBI‐GEO Database: two datasets were identified (GSE27562, GSE47862) (252 breast cancer patients and 194 healthy women)	Peripheral blood mononuclear cells (PBMC) (Genomic data)	SVIP, BEND3, MDGA2, LEF1‐AS1, PRM1, TEX14, MZB1, TMIGD2, KIT, FKBP7	ML	XGBoost
Silva‐Aravena et al. [82]	2023	Developing a clinical decision support methodology that performs early detection of BC and interprets the variables and how they affect patients' health	Public data on women from Indonesia [83] (400 anonymous patient cases, 200 of them with BC)	Text	High‐fat diet, breastfeeding	ML	XGBoost
Massafra et al. [84]	2023	Predict 5‐year and 10‐year breast cancer invasive disease events (IDEs)	(486 breast cancer patients) Breast and clinic research center IRCCS Istituto Tumori “Giovanni Paolo II” in Bari (Italy)	Clinical data	(5 years) Age, tumor diameter, surgery type, multiplicity. (10 years) therapy‐related features: hormone, chemotherapy schemes, lymphovascular invasion	ML	XGBoost
Vrdoljak et al. [85]	2023	Assessing metastatic lymph node status in BC patients eligible for neoadjuvant systemic therapy (NST)	Data collected from all Croatian hospitals (total study population (8381), NST‐criteria group (719))	Text	NST group: (tumor size, ER, PR, HER2), Total population: (tumor size, Ki‐67, tumor grade)	ML	NST‐criteria group (RF), Total study population → (XGBoost)
Uddin et al. [86]	2023	Investigate an ML model to forecast the development of BC more promptly	Breast Cancer Wisconsin (Diagnostic) [87]	Text	**Results for LightGBM:** perimeter_worst, concave points_mean, concave points_worst	ML	LightGBM, XGBoost, GBM
Zhao et al. [88]	2023	Predicting distant metastasis in male breast cancer (MBC) patients	2241 MBC patients from the SEER database between 2010 and 2015, and 110 MBC patients from a hospital between 2010 and 2020	Clinical and pathological TNM staging information data	T stage, age, N stage	ML	XGBoost
Cordova et al. [89]	2023	Classifying the epidermal growth factor 2 (HER2) photomicrographs to determine criteria that improve the value of immunohistochemical (IHC) analysis	393 histological slides of IHC‐stained breast cancer tissues from 2019 were randomly collected for the lab technician team of Carlos Van Buren Hospital Pathology Service (Valparaíso, Chile)	Microscopy images	**Results for IHC + FISH:** COUNT, MGV, M. SIZE, %AREA. **Results for IHC:** MGV, COUNT, M. SIZE, %AREA	ML	LR‐based to discriminate between upregulated and normal expression of HER2 protein

**Table 5:** List of studies that used local interpretable model agnostic explanations.

Authors	Year	Objective	Data set(s)	Data type	Important features	Machine learning (ML)/Deep learning (DL)	Explained model
Kaplun et al. [90]	2021	Extract complex features from cancer cell images and classify malignant and benign cancer cell images	BreakHis [91]	Microscopic images	Yellow highlighted segments in the image	DL	ANN (2‐layer feed forward neural network)
Saarela et al. [92]	2021	Comparing different feature importance measurements using linear (LR) and nonlinear (RF) classification ML models	Breast Cancer Wisconsin (Diagnostic) [87]	Text	L1‐LR → all except one (compactness 3) RF → nine features were significant	ML	L1 regularized LR, RF
Adnan et al. [93]	2022	Proposing a model in BC metastasis prediction that can provide personalized interpretations using a very small number of biologically interpretable features	Amsterdam Classification Evaluation Suite (ACES) [94] (composed of 1616 patients, among which 455 is metastatic)	Genomic data	N/A	M/DL	RF, LR, lSVM, rSVM, ANN
Maouche et al. [95]	2023	Propose an explainable approach for predicting BC distant metastasis that quantifies the impact of patient and treatment characteristics	Public data set composed of 716 Moroccan women diagnosed with breast cancer [96]	Clinicopathological data	The characteristics have different impacts ranging from high, moderate, and low	ML	Cost‐sensitive CatBoost
Deshmukh et al. [97]	2023	Improve the qk‐means clustering algorithm using LIME to explain the predictions	The breast cancer data set has 600 attributes or patient records and 7 features	Text	A tabular explainer explains the positively and negatively correlated features	ML	qk‐means (hybrid classical‐quantum clustering approach)

**Table 6:** List of studies that used class activation map.

Authors	Year	Objective	Data set(s)	Data type	Machine learning (ML)/Deep learning (DL)	Explained model
Qi et al. [98]	2019	Improving the efficiency and reliability of BC screening and guiding pathological examination by automating ultrasonography image diagnosis	Department of Galactophore Surgery and Department of Oncology of West China Hospital, Sichuan University (Over 8000 images from 2047 patients from October 2014 to August 2017)	Ultrasound	DL	Convolutional neural network (CNN)
Zhou et al. [99]	2019	Predicting clinically negative axillary lymph node metastasis from images in patients with primary breast cancer	Tongji Hospital (974 images (2016 to 2018)), independent test set (Hubei Cancer Hospital (81 imaging (2018 to 2019))	Ultrasound	DL	Inception V3
Huang et al. [100]	2020	Propose unsupervised DL learning model for medical image classification	CBIS‐DDSM: Breast Cancer Image Data set	X‐ray	DL	Modified PCANet (An unsupervised CNN model), DenseNet
Xi et al. [101]	2020	Proposing a DL‐based approach for abnormality detection in medical images	(1) Mammographic Image Analysis Society (MIAS). (2) Digital Database for Screening Mammography (DDSM)	X‐ray	DL	CNN
Kim et al. [102]	2021	Developing a weakly‐supervised CNN algorithm to diagnose breast cancer without using image annotation	1400 US images for breast masses of 971 patients from two institutions	Ultrasound	DL	VGG‐16, ResNet34, GoogLeNet

**Table 7:** List of studies that used Grad‐class activation map.

Authors	Year	Objective	Data set(s)	Data type	Machine learning (ML)/Deep learning (DL)	Explained model
Adoui et al. [103]	2020	Predicting the breast cancer response to Neoadjuvant chemotherapy (NAC) based on multiple MRI inputs	Institute of Radiology in Brussels (A cohort of 723 axial slices extracted from 42 breast cancer patients who underwent NAC therapy)	MRI	DL	Based on convolutional neural network (CNN)
Hussain et al. [104]	2022	Developing DL multiclass shape‐based classification framework for the tomosynthesis of breast lesion images	Based on the previous study [105]	Digital breast tomosynthesis (DBT)	DL	VGG, ResNet, ResNeXt, DenseNet, SqueezeNet, MobileNet‐v2
Agbley et al. [106]	2023	Breast tumor detection and classification using different magnification factors on the Internet of Medical Things (IoMT)	BreakHis [91]	Microscopic images	DL	ResNet‐18, Federated Learning (FL) to preserve the privacy of patient data
Gerbasi et al. [107]	2023	Proposing a fully automated and visually explained model to analyze raw mammograms with microcalcifications	INbreast data set [108] (train and test), CBIS‐DDSM [109] (used to implement the classification algorithm)	Scanned film Mammography	DL	U‐Net, ResNet18

**Table 8:** List of studies that used Grad‐class activation map++.

Authors	Year	Objective	Data set(s)	Data type	Machine learning (ML)/Deep learning (DL)	Explained model
To et al. [110]	2023	Improving classification performance and effectively identifying cancerous regions in DUV whole‐slide images (WSI)	Medical College of Wisconsin (MCW) tissue bank [111] (60 samples, 24 normal/benign and 36 malignant)	DUV‐WSI image	ML/DL	ResNet50, XGBoost

**Table 9:** List of explainable artificial intelligence studies that used layerwise relevance propagation.

Authors	Year	Objective	Data set(s)	Data type	Machine learning (ML)/Deep learning (DL)	Explained model
Grisci et al. [112]	2021	Propose relevance aggregation approach, a DL algorithm that correctly identifies which features are the most important for the network's predictions in an unstructured tabular data set	Curated Microarray Database (CuMiDa) [113]	Tabular unstructured data	DL	LSTM
Chereda et al. [114]	2021	Extend the procedure of LRP to make it available for Graph‐CNN (GCN) and test its applicability on a large breast cancer data set	Gene Expression Omnibus (GEO) [115]	Genomics data	DL	Graph‐CNN

## 5 DISCUSSION

### 5.1 Post hoc XAI methods: Model‐agnostic

#### 5.1.1 SHapley Additive exPlanations (SHAP)

SHAP [116] offer local and global explanations based on the Shapley value [117], a solution concept used in cooperative game theory. In SHAP, the input features of an observation act as players in a game, and the prediction serves as the reward. SHAP computes the average marginal contribution of each player to the reward [64, 65] and ensures that the distribution of reward among players is fair [18, 118]. In BC studies, SHAP can potentially find the contribution of biomarkers (players → important features in Table 4) to the prediction (reward  objective in Table 4).

As provided in Table 4, most studies (12/13) implemented ensemble ML learning as the predictors. Only in one study (1/13) [89] did authors first utilize LR‐based to discriminate between upregulated and regular expression of HER2 protein, then pathologists' diagnoses (IHC) in conjunction with fluorescent in situ hybridization (IHC + FISH) were used as the training outputs. In Chakraborthy et al. [74], SHAP showed that “by boosting the B cell and CD$8^{+}$ T cell fractions or B cell and NK T cell fractions in the tumor microenvironment (TME) to levels above their inflection points, the survival rate of BC patients could increase by up to 18%.” In Rezazadeh et al. [76], texture analysis of the ultrasound images based on the gray‐level co‐occurrence matrix (GLCM) predicted the likelihood of malignancy of breast tumors. SHAP was used to find the most critical features: GLCM correlation and GLCM energy within different pixel distances along the 90° direction.

In summary, SHAP emerged as the most frequently used XAI method in the BC studies (13/30). Notably, no DL models were used in conjunction with SHAP. Instead, tree‐based ensemble learning ML models, specifically XGBoost (9/13 studies), were the most widely used models. This can be attributed to the high‐speed SHAP algorithm, which is well‐suited for tree‐based models such as XGBoost, Catboost, GBM, AdaBoost, and so on [18].

#### 5.1.2 Local interpretable model agnostic explanations (LIME)

LIME [119] provides a local explanation using a surrogate model. As outlined in Table 5, LIME is utilized in 5 out of 30 studies to explain the model prediction by highlighting the contribution of the most important features. LIME creates a linear local surrogate mode that is intrinsically interpretable around a sample (data point) and improves transparency by producing feature importance values. The surrogate model in LIME modifies some parts of the given features and generates perturbed instances to understand how the output changes. The perturbation depends on the nature of the input sample. For instance, one method to perturb an image is by replacing certain parts with gray color [120]. In Kaplun et al. [90], to explain the image classification, LIME puts a mask of yellow pixels to highlight the important image segments the model focuses on to make the decision.

In Adnan et al. [93], the authors have implemented SHAP in conjunction with LIME to explain that a small number of highly compact and biological gene cluster features resulted in similar or better performance than classifiers built with many more individual genes. With training on smaller gene clusters, LIME proved that the classifiers have better AUC than the original classifiers except in RF and rSVM. In Saarela and Jauhiainen [92], the authors used linear and nonlinear ML classifiers with LIME to understand how they differ in explaining features' importance. It was found that the nonlinear model (RF) offered better explainability as it focused on fewer features (9) compared to the nonlinear model (all except one feature). Deshmukh et al. [97] used LIME to quantify the impact of patient and treatment characteristics on BC distant metastasis. It reached the results of different impacts ranging from high impacts, such as the nonuse of adjuvant chemotherapy, to moderate impact of carcinoma with medullary features cancer type, to a low impact of oral contraception use.

As a model‐agnostic method, LIME was used to explain various ML models, including RF, SVM, ensemble learning, and a shallow DL learning model, as detailed in Kaplun et al. [90]. LIME only offers a local interpretation, and compared to SHAP, when a large volume of predictions needs to be explained, it has a higher speed and can be a more excellent alternative. In summary of the model‐agnostic methods used in studies (18/30), SHAP was preferred over LIME to shed light on the most important features. This is because SHAP is relatively easy to implement and provides both local and global explanations, and compared to LIME, it has a higher speed on the global‐level explanation for high‐performance ensemble ML models.

### 5.2 Post hoc XAI methods: Model‐specific

Several XAI methods are specifically designed for different DL architectures focusing on the feature importance type of explanation. Most of these methods are propagation‐based and enjoy the availability of gradients computed during the training.

#### 5.2.1 Class activation map (CAM)

CAM [121] is a local backpropagation‐based method that uses a global average pooling (GAP) layer after the last convolutional layer, followed by the classification layer to identify the most discriminative regions of an image in the convolutional neural network (CNN) [34]. This technique combines a linear weighted sum of the feature maps to gain a heatmap that highlights class‐specific regions of the image. CAM is limited to existing networks that have the described architecture.

As listed in Table 6, (5/30) studies have used CAM to determine how accurately the CNN model localized the breast masses. Qi et al. [98] proposed two CNN‐based networks, the Mt‐Net and the Sn‐Net, to identify malignant tumors and recognize solid nodules step‐by‐step. To enable the two networks to collaborate effectively, CAM was introduced as an enhancement mechanism to improve the accuracy and sensitivity of the classification results for both networks.

#### 5.2.2 Gradient‐weighted class activation mapping (Grad‐CAM)

Grad‐CAM [122] is a local backpropagation‐based method that uses the feature maps produced by the last layer of a CNN to create a coarse localization class‐specific heatmap where the hot part corresponds to a particular class. Grad‐CAM is based on CAM but is not limited to fully connected CNNs. Grad‐CAM can be applied to any CNN architecture without retraining or architectural modification as long as the layers are differentiable.

As detailed in Table 7, (4/30) studies used Grad‐CAM to determine how accurately the CNN model localized the breast masses. The authors of article [104] have also investigated the performance of the model‐agnostic LIME in conjunction with Grad‐CAM to investigate the aspects and utilities of two different XAI methods in explaining the misclassification of breast masses. The results highlight the usability of XAI in understanding the mechanism of used AI models and their failures, which can provide valuable insights toward explainable CAD systems. In Gerbasi et al. [107], the authors also implemented Deep SHAP, a high‐speed approximation algorithm for computing SHAP values in DL models, to produce maps visually interpreting the classification results, which in the maps, pink pixels strongly contributed to the final predicted class (malignant), and the blue pixels contributed to the prediction of opposite class (benign).

#### 5.2.3 Gradient‐weighted class activation mapping++ (Grad‐CAM++)

Grad‐CAM++ [123] is a local backpropagation‐based method built upon Grad‐CAM to enhance visual explanations of CNN. Compared to Grad‐CAM, it provides better visual explanations of model predictions in terms of better localization of objects and explaining occurrences of multiple objects of a class in a single image [123]. As listed in Table 8, only one study (1/30) used Grad‐CAM++. To et al. [110] developed an ensemble learning‐based approach to locate cancerous regions in DUV whole‐slide images (WSI). It used Grad‐CAM++ on a pretrained DenseNet169 model to generate regional significance maps to classify each WSI confidently as cancerous or benign.

#### 5.2.4 Layer‐wise relevance propagation (LRP)

LRP [124] is a local propagation‐based approach. LRP calculates the relevance score for a specific output at the classifier layer. It proceeds backward, exploits the DL structure, and calculates each neuron's explanatory factors (relevance R) for each layer during the backward pass until it reaches the input image [18, 124]. Based on the computed relevance score, LRP generates a heatmap with highlighted critical regions that can be used to explain the prediction. Two studies (2/30) have implemented LRP; the details are described in Table 9.

Grisci et al. [112] introduced relevance aggregation, an XAI approach based on LRP that combines the relevance derived from several samples as learned by a neural network and generates scores for each input feature. The study results showed that the model could correctly identify which input features or relevant ones are the most important for the model's predictions, facilitate knowledge discovery, and help identify incorrect or irrelevant rules or machine biases in the case of the poorly trained implemented AI model. Chereda et al. [114] extended the procedure of LRP to make it available for GCN to explain its decisions. They tested it on a large BC genomic data set. They showed that the model, named graph layer‐wise relevance propagation (GLRP), provides patient‐specific molecular subnetworks that agree with clinical knowledge and can identify common, novel, and potentially druggable drivers of tumor progression.

In summary, 12 out of 30 studies used model‐specific XAI methods, and CAM and Grad‐CAM were the most used models, respectively.

### 5.3 Clinical applications

**Figure 6:**
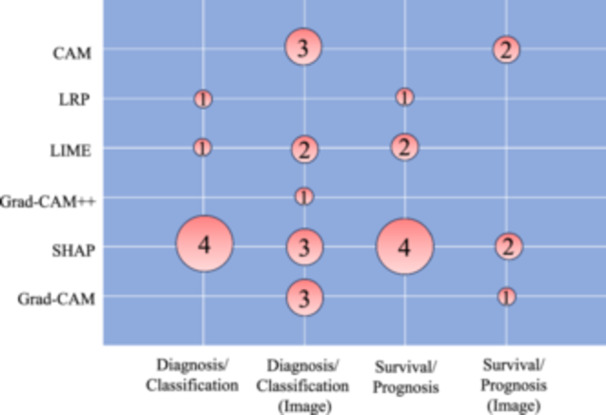
Explainable artificial intelligence (XAI) methods are applied in different clinical applications of breast cancer literature. Numbers inside the bubbles represent the number of studies.

Figure 6 illustrates the diverse applications of each XAI method across various clinical scenarios. The studies primarily focused on either diagnosing/classifying breast cancer or conducting survival/prognosis analyses of patients. Within these study types, image recognition techniques were employed on radiology data, or alternative approaches utilizing clinical and demographic data were explored. Notably, SHAP was frequently utilized in clinical data analysis studies rather than image recognition studies. This preference may be attributed to the computational resource intensity of SHAP, posing challenges in handling the high‐dimensional feature space inherent in image data. Conversely, techniques such as CAM and Grad‐CAM are computationally less intensive, which makes them a better choice for image processing tasks, especially in real‐time applications. In diagnosis/classification studies, the primary objective was to employ supervised learning methods for distinguishing between healthy and diseased patients, facilitating early detection. XAI models played a crucial role in helping clinicians comprehend and validate intricate patterns and features that influence diagnostic outcomes. In survival/prognosis models, clinicians sought to predict the onset of events such as mortality or metastasis in patients. XAI methods proved instrumental in interpreting and elucidating the contribution of each factor to a patient's outcome measure. This interpretability makes the models more understandable, usable, and trustworthy for both clinicians and patients, fostering a perception and interpretation of the predictions and building confidence in the decision‐making process.

### 5.4 Future directions

The rapid evolution of AI models, as evidenced by advanced frameworks such as GPT and generative AI‐based models, is significantly transforming the healthcare applications landscape. As these models continue to advance and become more intricate, the necessity for XAI methods becomes increasingly imperative. In the healthcare domain, where precision and interpretability are of paramount importance, the demand for robust XAI techniques is expected to grow. Future research should prioritize the refinement and advancement of XAI methodologies to effectively uncover the intricacies of advanced AI models in healthcare contexts. The synergy between the rapid advancements in AI technologies and the evolving landscape of XAI is crucial, shaping the trajectory of personalized healthcare and ensuring that these innovative models translate into tangible benefits for both clinicians and patients.

### 5.5 Study limitations

This systematic scoping review has some limitations that warrant consideration. While efforts were made to minimize publication bias, excluding non‐English language articles, non‐access, and gray literature may have resulted in the omission of some valuable information. Additionally, despite our best efforts to construct a comprehensive search strategy across multiple databases using combinations of Boolean search strings and MeSH terms, the diverse terminology associated with XAI methods and breast cancer may have led to the inadvertent omission of certain studies. Moreover, we only investigated the existing established XAI methods; however, XAI schemes based on or independent of these methods could be observed in a few studies. To ensure the integrity and credibility of the study, we did not consider some of the studies with low or no citations in this survey.

## 6 CONCLUSIONS

We systematically reviewed breast cancer studies that successfully implemented the existing XAI methods to their model predictors. In summary, SHAP was the most used model‐agnostic method. The frequent use of this method with tree‐based ensemble ML models is related to the speed and compatibility that SHAP provided for these models. Grad‐CAM and CAM were widely used model‐specific XAI methods in these studies. We noticed that other explanatory methods, as provided in Appendix A, have not been used in breast cancer studies and can still be examined and compared as future endeavors.

Additionally, the XAI methods used in the selected studies only provided a sanity check to the model's predictor results. As was mentioned in the introduction, finding the biases in the model and data can be achieved using explainability methods that were either missing or only mentioned in a few of the studies and should be investigated for further studies. Moreover, although the clinical applications of XAI methods were investigated in our study, the results generated by these methods were not evaluated by oncologists. Therefore, to provide trustworthiness, the reliability of the results through clinical evaluation is needed. Researchers have already used XAI domain‐specific explanations to improve understanding, interpretation, trustworthiness, and reliability of the results in different medical domains for evaluating health interventions [125], disease causal pathway analysis [126], mental health surveillance and precision resource allocation [127], precision dermatology and disease diagnosis [128], immune response predictors [129], and investigating the links between socioenvironmental risk factors and Alzheimer's disease [130].

Potential challenges associated with the application of XAI, especially when dealing with complex multimodal clinical and medical data, include but are not limited to the availability of data in appropriate temporal and geographic resolutions; its representativeness, diversity, and types of modalities involved, semantic heterogeneity, fusion of heterogeneous data streams, AI‐readiness of clinical data sets [131], and algorithmic and human biases in explanations that addressing them can increase the efficiency and acceptance of multimodal XAI schemes.

Addressing these challenges is key to the widespread acceptance of multimodal XAI models and algorithms in cancer care delivery and treatment.

## AUTHOR CONTRIBUTIONS

**Amirehsan Ghasemi**: Conceptualization (lead); data curation (lead); formal analysis (lead); investigation (lead); methodology (lead); validation (lead); visualization (lead); writing—original draft (lead); writing—review and editing (lead). **Soheil Hashtarkhani**: Conceptualization (supporting); data curation (supporting); formal analysis (supporting); investigation (supporting); methodology (supporting); validation (supporting); visualization (supporting); writing—original draft (supporting); writing—review and editing (supporting). **David L. Schwartz**: Conceptualization (supporting); funding acquisition (supporting); investigation (supporting); resources (supporting); supervision (supporting); writing—original draft (supporting); writing—review and editing (supporting). **Arash Shaban‐Nejad**: Conceptualization (lead); funding acquisition (lead); methodology (supporting); project administration (lead); resources (lead); supervision (lead); writing—original draft (supporting); writing—review and editing (supporting).

## CONFLICT OF INTEREST STATEMENT

The authors declare no conflict of interest.

## ETHICS STATEMENT

Not applicable.

## INFORMED CONSENT

Not applicable.

## Data Availability

The authors declare that the data supporting the findings of this study are available within the paper.

## APPENDIX A POPULAR MODEL‐AGNOSTIC AND MODEL‐SPECIFIC XAI METHODS

The summary of model‐agnostic and model‐specific XAI methods is listed as follows.

See Tables A1, A2, A3, A4.

**Table A1:** Model‐agnostic explainable artificial intelligence (XAI) methods.

Model‐agnostic method	Acronym	Type of XAI	Scope of XAI	Technique
SHapley Additive exPlanations [116]	SHAP	Feature importance	Local, Global	Game‐theory
Local interpretable model agnostic explanations [119]	LIME	Feature importance	Local	Surrogate model
Anchors [132]	N/A	Feature importance	Local	Surrogate model
Occlusion sensitivity [133]	Occlusion	Feature importance	Local	Perturbation‐based
Partial dependence plots [134]	PDP	Visual explanations	Global	Marginalization
Counterfactuals [135]	N/A	Example‐based XAI	Global	Data point
Rule extraction [66]	N/A	White‐Box model	Global	White‐box
Tree extraction [67]	N/A	White‐Box model	Global	White‐box

**Table A2:** Model‐agnostic: Code/Toolbox.

Model‐agnostic method	Code/Toolbox
SHAP	https://github.com/slundberg/shap
LIME	https://github.com/marcotcr
Anchors	https://github.com/marcotcr/anchor
Occlusion	Can be found here: https://www.mathworks.com
PDP	Can be found here [136]
Counterfactuals	Can be found here [137]→ https://github.com/SeldonIO/alibi

**Table A3:** Model‐specific explainable artificial intelligence (XAI) methods.

Model‐specific method	Acronym	Black‐box model	Type of XAI	Scope of XAI	Technique
Class activation map [121]	CAM	Convolutional neural network (CNN)	Feature importance	Local	Propagation‐based
Gradient‐weighted class activation mapping [122]	Grad‐CAM	CNN	Feature importance	Local	Propagation‐based
Gradient‐weighted class activation mapping++ [123]	Grad‐CAM++	CNN	Feature importance	Local	Propagation‐based
Integrated gradients [138]	IG	All DL models	Feature importance	Local	Propagation‐based
Deep Learning Important FeaTures [139]	DeepLIFT	All DL models	Feature importance	Local	Propagation‐based
Layerwise relevance propagation [124]	LRP	All DL models	Feature importance	Local	Propagation‐based
Deep Taylor decomposition [140]	DTD	All DL models	Feature importance	Local	Propagation‐based
Guided backpropagation [141]	GBP	All DL models	Feature importance	Local	Propagation‐based
Activation maximization [142]	N/A	All DL models	Feature importance	Global	Propagation‐based
Testing with concept activation vectors [143]	TCAV	All DL models	Feature importance	Global	Concept‐based
Model explanation for graph neural networks [144]	GraphLIME	GNN	Feature importance	Local	Surrogate model

**Table A4:** Model‐specific explainable artificial intelligence methods: Code/Toolbox.

Model‐specific method	Code/Toolbox
CAM	https://github.com/zhoubolei/CAM
Grad‐CAM	https://github.com/ramprs/grad-cam/
Grad‐CAM++	https://github.com/adityac94/Grad_CAM_plus_plus
IG	https://github.com/ankurtaly/Integrated-Gradients
DeepLIFT	https://github.com/kundajelab/deeplift
LRP, DTD	https://github.com/chr5tphr/zennit
	https://github.com/albermax/innvestigate
Guided backpropagation	https://github.com/mateuszbuda/ALL-CNN
TCAV	https://github.com/tensor?ow/tcav
GraphLIME	https://github.com/WilliamCCHuang/GraphLIME
